# Understanding the basis of a novel fruit type in Brassicaceae: conservation and deviation in expression patterns of six genes

**DOI:** 10.1186/2041-9139-3-20

**Published:** 2012-09-03

**Authors:** Mariano Avino, Elena M Kramer, Kathleen Donohue, Alexander J Hammel, Jocelyn C Hall

**Affiliations:** 1 Department of Biological Sciences, University of Alberta, Edmonton, AB, T6G 2E9, CANADA; 2 Department of Organismal and Evolutionary Biology, Harvard University, Cambridge, MA, 02138, USA; 3 Department of Biology, Duke University, Durham, NC, 27708, USA

**Keywords:** *ALCATRAZ*, Brassicaceae, Fruit development, *INDEHISCENT*, *FRUITFULL*, *REPLUMLESS*, *SHATTERPROOF*, Silique

## Abstract

**Background:**

Variation in fruit morphology is important for plant fitness because it influences dispersal capabilities. Approximately half the members of tribe Brassiceae (Brassicaceae) exhibit fruits with segmentation and variable dehiscence, called heteroarthrocarpy. The knowledge of the genetics of fruit patterning in Arabidopsis offers the opportunity to ask: (1) whether this genetic pathway is conserved in taxa with different fruit morphologies; (2) how the pathway may be modified to produce indehiscence; and (3) whether the pathway has been recruited for a novel abscission zone.

**Methods:**

We identified homologs of *ALCATRAZ*, *FRUITFULL*, *INDEHISCENT*, *SHATTERPROOF*, and *REPLUMLESS* from two taxa, representing different types of heteroarthrocarpy. Comparative gene expression of twelve loci was assessed to address how their expression may have been modified to produce heteroarthrocarpy.

**Results:**

Studies demonstrated overall conservation in gene expression patterns between dehiscent segments of *Erucaria erucarioides* and Arabidopsis, with some difference in expression of genes that position the valve margin. In contrast, indehiscence in heteroarthrocarpic fruit segments was correlated with the elimination of the entire valve margin pathway in *Erucaria* and *Cakile lanceolata* as well as its absence from a novel lateral abscission zone.

**Conclusions:**

These findings suggest that modifications in the valve margin positioning genes are responsible for differences between heteroarthrocarpic and Arabidopsis-like fruits and support the hypothesis that heteroarthrocarpy evolved via repositioning the valve margin. They also highlight conservation in the dehiscence pathway across Brassicaceae.

## Background

In evolutionary developmental genetic studies, flowers have largely overshadowed fruits despite the fact that fruits display the same diversity in form (reviewed in [1]). Variation in fruit morphology is fundamentally tied to plant dispersal, a key component of angiosperm fitness. Not only have fleshy, dry dehiscent, and dry indehiscent fruits evolved multiple times across flowering plants [2,3], differences in fruit morphology are also observed at close phylogenetic distances within families. For example, berries have evolved more than once in both Solanaceae [4] and Melastomataceae [5]. Even families that have unique fruit types such as Fabaceae (legume) or Brassicaceae (silique) display considerable variation in form, segmentation, shape, and dehiscence capabilities [6-9]. Here we investigate the basis of fruit diversity within the Brassicaceae, the family that houses the genetic model *Arabidopsis thaliana*.

The genetic basis of dry dehiscent fruits has been thoroughly investigated in Arabidopsis (reviewed in [10-12]), providing a valuable framework for comparative studies of other brassicaceous fruits. Arabidopsis has a typical silique (hereafter referred to as typical silique), which is a bicarpellate capsule with two valves that dehisce or open at maturity. The valves surround the entire ovary and eventually separate from persistent placental tissue (replum) along the valve margin. The replum is connected by a thin membranous septum that separates the ovary into two chambers. The valve margin comprises two layers: a thick line of cells adjacent to the replum that form the separation layer, and a group of lignified cells adjacent to the valve that form the lignified layer. The valves detach through the enzymatic breakdown of the middle lamella of the cells in the separation layer, aided by the mechanical tension of lignified cells in the lignified layer and in the endocarp *b* (end*b*) layer of the valve [13]. Thus, fruit opening in Arabidopsis is dependent on the proper positioning and formation of the valve margin and its dehiscent zone (DZ).

Recent genetic studies on fruit development in Arabidopsis have emphasized the patterning and positioning of the valve margin. The valve margin and its subsequent DZ are determined by the combined activities of *SHATTERPROOF1* (*SHP1*), *SHATTERPROOF2* (*SHP2*), *INDEHISCENT* (*IND*), and *ALCATRAZ* (*ALC*), which represent a genetic pathway [11,14]. The closely related MADS-box containing paralogs *SHP1*/*2* are both expressed in the ovules, septum, and valve margin [15,16]. *SHP1/2* are functionally redundant and their double mutant phenotype exhibits a loss of valve margin identity with the resultant loss of the DZ [17]. *IND* and *ALC*, both encoding bHLH proteins, are genetically downstream of *SHP1/2*[11,14]. *IND* is the more pleiotropic of the two, specifying the identity of both the separation and lignified cell layers of the valve margin [14]. *ALC* plays a more limited role in the differentiation only of the separation layer [18]. The MADS-box gene *FRUITFULL* (*FUL*) and the BEL1-class homeodomain gene *REPLUMLESS* (*RPL*) are required to restrict *SHP1/2*, *ALC*, and *IND* to the narrow strip of cells at the valve/replum boundary [14,19,20]. *FUL* is expressed in the valves whereas *RPL* is expressed in the replum. Thus, the two genes play complementary roles to restrict the development of the valve margin identity to a thin region of cells via the repression of the *SHP1/2*, *IND*, and *ALC*[14,20]. *RPL* repression of *SHP1/2* in the valve margin is likely mediated by limiting the expression of *JAGGED* (*JAG*), *FILAMENTOUS FLOWER* (*FIL*), and *YABBY3* (*YAB3*), which in turn promote *FUL* and *SHP1/SHP2* expression [11]. Both *FUL* and *RPL* are valve margin positioning genes. Whereas *FUL* is not required to induce valve formation, evidence indicates that *RPL* contributes to replum formation [21-23]. Studies in *Brassica*, a member of Brassicaceae with fruits similar to Arabidopsis, demonstrate that the roles of *FUL*, *IND*, and *RPL* in this pathway have been conserved [23-25], however the entire pathway has not been examined in any indehiscent relative.

**Figure 1:**
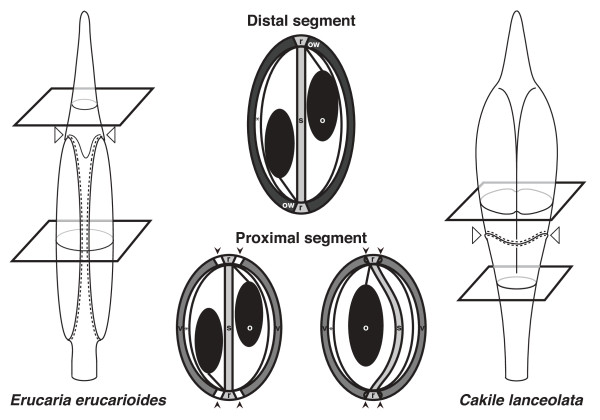
**Diagrammatic representation of fruits of focal species.** Left panel: *Erucaria erucarioides* fruit in medial view. Right panel: *Cakile lanceolata* fruit in medial view. Central panel, top: transverse section through the distal segments of *Erucaria* and *Cakile*. Central panel, bottom: transverse sections through proximal segment of *Erucaria* (left) and *Cakile* (right). White arrowheads in left and right panels indicate the position of the joint, black arrowheads in the central panel indicate the position of the valve margin, dotted lines represent abscission zones at fruit maturation. o, ovules; ow, ovary walls; r, replum; s, septum; v, valves; *, endocarp *b* layer.

The monophyletic tribe Brassiceae is characterized by a novel fruit type [26-28] that appears to be the product of, at least in part, modifications in the position of the valve margin [29], thus offering an excellent opportunity to examine how changes in this genetic pathway may lead to important differences in fruit morphology. Approximately 40% of the genera in Brassiceae have heteroarthrocarpic fruits, which are bisected by a joint that may or may not abscise at maturity [30]. The joint represents the distal portion of the valve margin, which has been elaborated via internal proliferation of the mesocarp [29], and completely bisects the fruit laterally thus running throughout the medio-lateral axis. Thus, in heteroarthrocarpic fruits the valves no longer surround the entire ovary as observed in Arabidopsis. In fact, only the ovary wall of the proximal (lower) segment differentiates into valve (Figure 1). This proximal segment varies in dehiscence depending on the species, but is valvular in origin regardless of whether or not it dehisces at maturity [29-31]. In contrast, the distal segment is invariably indehiscent. Anatomical differences between the proximal and distal segments indicate that the distal segment is indehiscent because the ovary wall does not differentiate into valves in this portion of the fruit (Figure 1) [29]. In other words, although the replum and septum are present throughout the ovary of heteroarthrocarpic fruits, there is no longer juxtaposition between valves and replum in the distal segment. This disassociation between valves and replum in heteroarthrocarpic fruits when compared to typical siliques is due to the position of the valve margin differing between these fruit types. However, it is clear that other modifications are needed for the transition from a typical silique to heteroarthrocarpy [29]. For example, the joint represents a novel abscission zone when it breaks the fruit into two segments, a phenomenon commonly referred to as disarticulation. 

Heteroarthrocarpy has important implications for dispersal because indehiscence creates dispersal propagules that are protected by the pericarp, and the joint results in seeds from the same plant being passively dispersed in separate and often morphologically different propagule types [32-34]. Moreover, variants of heteroarthrocarpy reflect differences in dehiscence capabilities. Here we focus on two closely related species, *Erucaria erucarioides* and *Cakile lanceolata*, that display different types of heteroarthrocarpy [28,29]. The proximal segment of *Erucaria* shows valve dehiscence but the joint itself does not abscise (Figure 1). As such, the seeds in the proximal segment are dispersed freely into the environment when the valves open, in essentially the same manner as observed in Arabidopsis, whereas seeds in the distal segment are dispersed in their enclosing pericarp often when the maternal plant dies. In contrast, *Cakile* has heteroarthrocarpic fruits that are completely indehiscent with a joint that abscises at maturation, separating the fruit into two segments (Figure 1). Although all the seeds in the fruit are dispersed enclosed in pericarp, the distal segment is freely dispersed when the joint abscises, while seeds contained in the proximal segment frequently remain attached to the maternal plant even after its death.

To understand the genetic basis of heteroarthrocarpy and variation in dehiscence, we isolated homologs of all major genes in the valve margin genetic pathway of Arabidopsis from *Cakile* and *Erucaria* and compared gene expression patterns throughout flower and fruit development. Examination of these taxa permits comparisons within fruits as well as between species. Furthermore, focusing on these two species allowed us to simultaneously explore the extent to which expression patterns in the genetic pathway of Arabidopsis have been conserved with regards to dehiscence as well as how they may be modified in species with indehiscent and segmented fruits. We expected to observe similar expression patterns in members of the genetic pathway between the dehiscent segment of *Erucaria* and Arabidopsis, due to the anatomical similarities [29]. We also predicted the loss of expression of at least some of the valve margin pathway genes in the ovary wall of indehiscent segments. However, differences between the distal and proximal segments were expected because the proximal segment of *Cakile* is valvular in origin in contrast with the distal segments of both species [29]. We had no *a priori* predictions where the pathway may be disrupted in indehiscent segments because dehiscence in Arabidopsis can be lost by loss of function mutations in all genes of the valve margin pathway as well as overexpression of *FUL* (reviewed in [12]). We hypothesized that the valve margin pathway might have been recruited to pattern the novel dehiscence zone in the joint of *Cakile* because the joint is anatomically similar to the DZ between the valve and replum [29,35]. Our data support conservation of gene expression patterns between dehiscent proximal segments of *Erucaria* and fruits of Arabidopsis, especially regarding the genes that promote valve margin identity. Notably, there was less conservation in the genes responsible for positioning the valve margin. Indehiscence was correlated with loss of gene expression of the entire valve margin genetic pathway in both the indehiscent, proximal segment of *Cakile* and the distal segments of both species. Surprisingly, the joint was also characterized by the absence of expression of valve margin genes. The implications of these patterns for the evolution of heteroarthrocarpy are discussed.

## Methods

### Plant material and growth conditions

Seeds of *Cakile lanceolata* (Willd.) O.E. Schulz and *Erucaria erucarioides* (Coss. and Durieu) MüllBerol were obtained from Russell Reardon (US Department of Commerce, National Oceanic and Atmospheric Administration, Florida Keys National Marine Sanctuary) and the late César Gómez-Campo’s seed collection, respectively. Species were grown from seed in the Harvard University greenhouses and the University of Alberta, Department of Biological Sciences growth chambers. Flowers of both focal taxa were hand pollinated to ensure seed set.

### Identification of valve margin pathway homologs

RNA was extracted from developing flowers and fruits using Concert Plant RNA Reagent kit (Invitrogen, Carlsbad, CA, USA), DNAse1 treated to remove DNA (Fermentas, Honover, MD, USA) and then cleaned with RNAeasy MiniKit (Qiagen, MD, USA). mRNA was purified from total RNA with the Dynabeads mRNA Purification Kit (Invitrogen, Karlsruhe, Germany). cDNA was then synthesized from the mRNA using the RevertAid H Minus First Strand cDNA Synthesis Kit (Fermentas, St Leon-Rot, Germany) or the SuperScriptII reverse transcriptase (Invitrogen, Carlsbad, CA, USA) by priming with polyT primer [36]. *ALC*-, *FUL*-, *IND*-, *RPL*-, and *SHP1/2*-like genes were amplified via PCR from *Erucaria* and *Cakile* cDNA. In order to establish copy number, we used a set of broadly degenerate primers that picked up other related loci and used multiple primer combinations (see Additional file 1: Table S1, for primer information and sequences). Primers were designed using mostly a nested primer design and by comparison to known sequences, particularly within *Arabidopsis thaliana*. PCR products were cloned into TOPO-TA (Invitrogen, Carlsbad, CA, USA). An average of 42 independent clones were sequenced per identified locus with a minimum number of 10 sequenced. Restriction analysis was also employed to screen colonies for potentially different copies.

Sequences were manually edited in MacVector v. 11.1 (MacVector Inc., Cary, NC, USA) and putative gene identity was initially established with BLASTn searches. To confirm gene homology, phylogenetic analyses were then conducted. Amino acid sequences were aligned with GenBank sequences (Additional file 2: Table S2) in either MacVector or BioEdit v.7.0.9.0 [37]. Neighbor-joining (NJ) trees were generated in MEGA v.5 [38]. As we are primarily interested in identifying homologs, not reconstructing phylogenies to infer relationships amongst genes, the NJ approach was sufficient to assess homology.

### *In situ* hybridization

*In situ* hybridization is the best available method for obtaining gene expression patterns in space and time [39], which is especially important when examining genes that potentially exhibit within-organ patterning (for example, within the presumptive valve margin of the carpel). Moreover, the candidate loci show a very close correspondence between their expression patterns and their function in Arabidopsis, although this may not hold true for *Cakile* and *Erucaria*. Developing inflorescences (including floral meristems), flowers and fruits from *Cakile* and *Erucaria* were collected and fixed under vacuum in freshly prepared, cold FAA (50% ethanol, 4% formalin, and 5% glacial acetic acid). We were unable to conduct *in situ* hybridization on mature fruits, as their heavily lignified tissue is not amenable to sectioning. After an incubation of 12 to 16 h, specimens were dehydrated in an ethyl alcohol series, embedded in paraplast, and stored at 4°C until use. Wax blocks were sectioned to 8μm on a Reichert-Jung (Buffalo, NY, USA) or a Microm HM 325 (Walldorf, Germany) microtome. DNA templates for RNA probe synthesis were obtained by PCR amplification of 150- to 500-bp fragments from cDNA clones of 12 loci: *ClALC*, *ClFUL1*, *ClFUL2*, *ClRPL*, *ClSHP1*, *ClSHP2*, *EeALC*, *EeFUL1*, *EeFUL2*, *EeIND*, *EeRPL*, and *EeSHP2* (Additional file 1: Table S1 for primer information). Probe templates included 100 to 250 bp of the 3′ untranslated region in addition to 50 to 250 bp from the 3′ coding region for gene specificity, except the *EeSHP2* and *ClSHP2* probes included only the 3′ coding region. Probes for recent paralogs, *SHP1/SHP2* and *FUL1/FUL2*, were designed in regions of highest variation between paralogs. Fragments were cloned using the TOPO-TA plasmid vector (Invitrogen, Carlsbad, CA, USA) and confirmed by sequencing. Both sense and antisense digoxigenin-labeled RNA (Roche, Indianapolis, IN, USA) probes were prepared from linearized template plasmids and alkaline hydrolyzed to 150 to 300 bp [40]. *In situ* hybridization was then performed following described methods [39]. Sections were visualized and imaged using a combination of white and fluorescent light after counterstaining with calcofluor. Sections were digitally photographed using a Leica Leitz DMRD microscope equipped with a Retiga EXi imaging system (Harvard University imaging center) or a NIKON H550L fluorescence microscope equipped with a Nikon DS-Ri1 imaging system (University of Alberta).

### Expression studies

Characterization of differential expression in floral organs of *Cakile* and *Erucaria* was performed using reverse transcription-polymerase chain reaction (RT‐PCR) on cDNA from the following tissue: (1) young buds (<4 mm), corresponding to carpels before differentiation into discernable replum, ovary wall/valve, and endocarp [29]; (2) old buds (>4 mm), corresponding to carpels after differentiation; (3) flowers at anthesis; (4) young fruits approximately 10 days post-fertilization (length: circa 1 cm); (5) mature fruits approximately 60 days (*Cakile*) and 25 days (*Erucaria*) post-fertilization (1.8 cm long per *Cakile*, 1.5 cm long per *Erucaria*); and (6) leaves. Fruits were not dissected between distal and proximal because both segments contain elements of the joint [29]. RNA was extracted using Concert Plant RNA Reagent kit (Invitrogen, Carlsbad, CA, USA) then DNAse treated to remove DNA (TURBO DNA-free kit, Ambion). cDNA was then synthesized from 2 μg RNA using SuperScriptIII reverse transcriptase (Invitrogen, Carlsbad, CA, USA). RT-PCR reactions were carried out using locus-specific primers (Additional file 1: Table S1), 1.0 U of *rTaq* or *exTaq* DNA polymerase (Takara Co., Tokyo, Japan) in 25 μL of PCR buffer (20 mM Tris‐HCl pH 8, 100 mM KCl, 0.1 mM EDTA, 1 mM DTT, 0.5% Tween®20, 0.5% Nonidet® P-40, 50% Glycerol) containing 30 pmol of 5′ and 3′ primers, 200 μM of each dNTP, and 0.5-1 μL of cDNA. Amplification program was as follows: 94° for 2 min, followed by 15 to 27 cycles of 94° for 20 s, 50° to 55° for 30 s, and 72° for 1 min. We established the linear range of amplification by testing different cycle durations. No bands were discerned for genes of interest at 15 cycles, weak bands were observed at 20 in *Cakile* (but not *Erucaria*), moderate bands at 22, and strong bands at 27. The 27 band results are shown in the text but the 20 and 22 are also shown in the supplement. Reactions were run on a 1.2% agarose gel and digitally photographed using Alpha Innotech’s Red™ Imaging System (San Leandro, CA, USA). The identity of amplified fragments was confirmed by amplicon size and sequencing.

## Results

### Identification of the valve margin pathway genes

To establish the possible roles of valve margin pathway genes in the development and evolution of heteroarthrocarpy, we identified homologs of *ALC*, *FUL*, *IND*, *RPL*, and *SHP1*/*SHP2* from *Cakile* (*Cl-*) and *Erucaria* (*Ee-*). Screening of putative orthologs revealed one copy of every gene except *FUL*, which had two copies in both focal species (GenBank: JX292959-JX292970). *SHP1* was not identified from *Erucaria* and *IND* was not found in *Cakile* cDNA. Thus, 12 loci were identified in total: *ClALC*, *ClFUL1*, *ClFUL2*, *ClRPL*, *ClSHP1*, *ClSHP2*, *EeALC*, *EeFUL1*, *EeFUL2*, *EeIND*, *EeRPL*, and *EeSHP2*. Gene identity was confirmed by separate phylogenetic analyses of *AGAMOUS*, *APETALA1/FUL*, bHLH, and BEL-like sequences from a range of angiosperms (Additional file 3: Figure S1, Additional file 4: Figure S2, Additional file 5: Figure S3, and Additional file 6: Figure S4, Additional file 2: Table S2). In all instances, the putative homologs from *Cakile* and *Erucaria* were closely related to genes from other Brassicaceae, specifically Arabidopsis and *Brassica* (for example, *ClALC* plus *EeALC* are sister to *ALC*; Additional file 3: Figure S1, Additional file 4: Figure S2, Additional file 5: Figure S3, and Additional file 6: Figure S4).

Although the tribe Brassiceae has experienced an ancient triploidization [41,42], only two duplications were observed in *Cakile* and *Erucaria* among the 12 *SHP-*, *FUL-*, *ALC-*, *IND*, and *RPL-*like genes identified. The Brassicaceae-specific duplication of *SHP1*/*SHP2*[43,44] was recovered in *Cakile*, but not *Erucaria*. Additional studies of *Erucaria* are needed to determine if this represents a real loss of *SHP1*. Importantly, two copies of *FUL* were uncovered in both focal taxa. These findings are somewhat consistent with that of *Brassica*, the only other taxon within the tribe for which sequence information is available. In *Brassica*, only one copy of *IND* is present [24], whereas multiple copies of *FUL*, *RPL*, and *SHP* are observed (Additional file 3: Figure S1, Additional file 4: Figure S2, Additional file 5: Figure S3, and Additional file 6: Figure S4; [23,45]).

### Valve margin pathway expression data in *Erucaria erucarioides*

**Figure 2:**
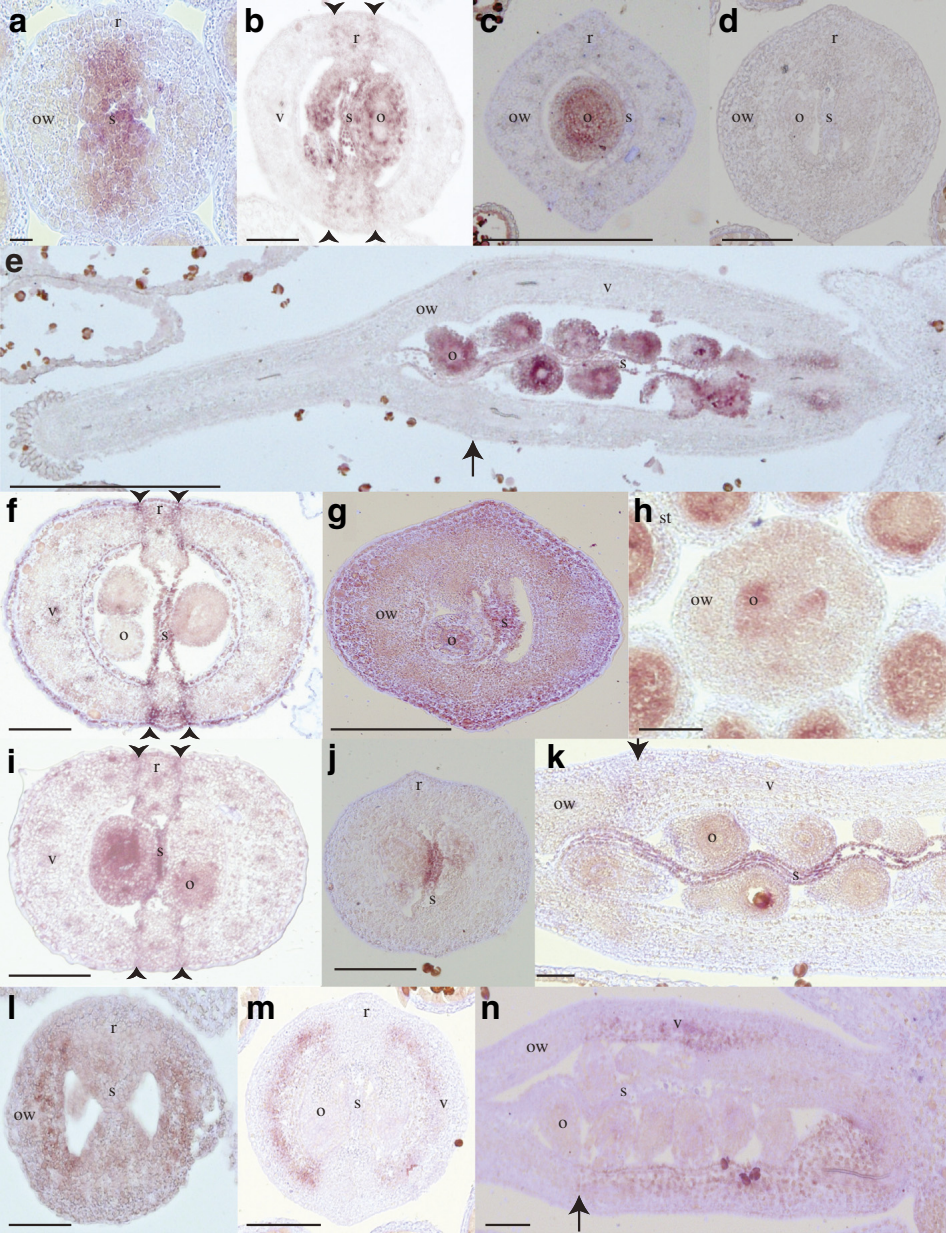
***In situ* hybridization expression of *EeSHP2*, *EeALC*, *EeIND*, and *EeFUL1* during *Erucaria erucarioides* carpel development. (a-c)** Transverse sections through buds showed *EeSHP2* expression. (**a**) Expression in the developing septum of a young bud. (**b**) Expression at a later stage in the septum, ovules, and valve margin of the proximal segment. (**c**) Expression in the ovules of the distal segment. (**d**) *EeSHP2* sense control in a transverse section. (**e**) Longitudinal section of a differentiated carpel showed *EeSHP2* expression in septum and ovules. (**f**) *EeALC* expressed in the valve margin, septum, end*b*, and exocarp of a proximal segment, transverse section of an old bud. (**g**) Signal of *EeALC* in the distal segment was detected in the septum of differentiated carpel, transverse section. (**h**) Transverse section of an undifferentiated carpel showed *EeIND* expression in ovule primordia. (**i**-**k**) *EeIND* was expressed in valve margin and ovules of the proximal segment of differentiated carpel, transverse section (**i**), but only septum and ovules of proximal as shown in transverse (**j**) and longitudinal (**k**) sections of differentiated carpel. (**l**) Transverse section showed *EeFUL1* expression throughout inner valves, but not replum or presumptive valve margin, of undifferentiated carpel. (**m**, **n**) *EeFUL1* was expressed in inner valves of the proximal segment, as seen in transverse (**m**), but not in the ovary walls of the distal segment of differentiated carpel, longitudinal (**n**). Arrows indicate the position of the joint, arrowheads indicate the position of the valve margin. o, ovules; ow, ovary walls; r, replum; s, septum; st, stamen; v, valves. Scale bar: 50 μm (**a**), 100 μm (**b**, **c**, **d**, **f**, **g**, **h**, **i**, **j**, **k**, **l**, **m**), 500 μm (**e**, **n**).

We used *in situ* hybridization to determine the spatial and temporal expression patterns of the valve margin pathway genes identified from *Erucaria*. *EeSHP2* expression was observed throughout floral and early fruit development. Before carpel differentiation, *EeSHP2* expression was detected in the central area of the carpel where ovules and septum will be formed, and extended throughout the forming repla (Figure 2a). Later in development, when layers of the mesocarp and valve margin had differentiated, the *EeSHP2* expression domain encompassed the ovules, with stronger signal in the inner integument, and the septum of both segments (Figure 2b, c, and e). In the ovary wall, *EeSHP2* expression was restricted to the presumptive valve margin of the proximal segments (Figure 2b). No *EeSHP2* expression was observed in the ovary wall of the indehiscent distal segment (Figure 2c) or with sense probes (Figure 2d). *EeALC* and *EeIND* have similar expression domains in developing flowers and fruits. Expression of *EeALC* was first observed in the forming septa of young buds (data not shown). Later in development, *EeALC* was detected in septa of both proximal (Figure 2f) and distal (Figure 2g) segments. In the proximal segment, the expression domain was expanded to include the valve margin, end*b*, and exocarp layers (Figure 2f). Before carpel differentiation, *EeIND* was expressed in the region of ovule formation (Figure 2h). Although *EeIND* was expressed in the septum and ovules of both the segments (Figure 2i to k), expression in the presumptive valve margin was only observed in the proximal segment (Figure 2i). In sum, *EeSHP2*, *EeIND*, and *EeALC* were all expressed in the presumptive valve margin of the proximal, but not the distal segment.

The expression domain of *EeFUL1* was restricted to the valves of developing flowers and fruits. Early in development, *EeFUL1* expression was observed in the inner tissue of developing valves (Figure 2l). After layers of the ovary wall had differentiated, *EeFUL1* had a specific expression domain in the inner layer of the mesocarp and the end*b* layer (Figure 2m), but only of the proximal segment (Figure 2n). Occasionally, expression of *EeFUL1* was also observed in the ovules and nectaries of young and old buds (Additional file 7: Figures S5a to b). *EeFUL2* had a markedly different expression profile than *EeFUL1* with expression observed in the ovules and nectaries of young and old buds, but not in the valves (Additional file 7: Figure S5c).

**Figure 3:**
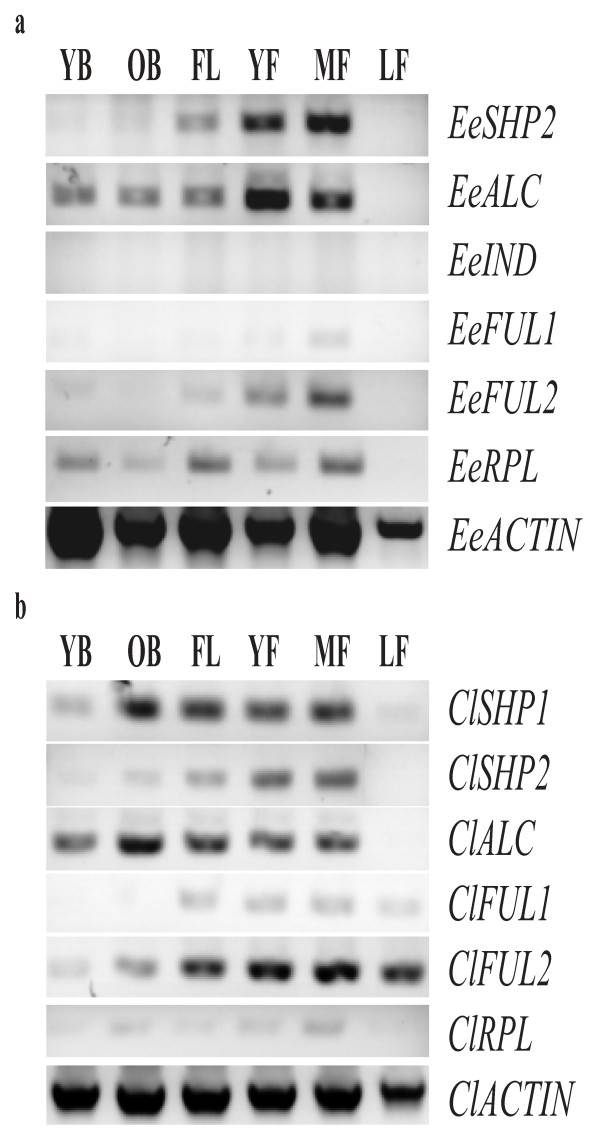
**Reverse transcriptase polymerase chain reaction.** RT-PCR expression analysis of (**a**) *EeSHP2*, *EeALC*, *EeIND*, *EeFUL1*, *EeFUL2*, *EeRPL*, *EeACTIN*, and (**b**) *ClSHP1*, *ClSHP2*, *ClALC*, *ClFUL1*, *ClFUL2*, *ClRPL*, and *ClACTIN* in young buds (YB), old buds (OB), flowers (FL), young fruits (YF), mature fruits (MF), and leaf tissue (LF).

We were unable to detect *EeRPL* expression after multiple attempts with different probes despite identifying the homolog from cDNA extracted from developing buds and fruits. To reconcile this contradiction, we complemented *in situ* hybridization with RT-PCR experiments on all identified loci (Figure 3). We used 27 cycles to assess expression patterns (Figure 3a; see Additional file 7: Figure S5d for lower cycle numbers). RT-PCR expression of *EeALC*, *EeFUL1*, *EeFUL2*, and *EeSHP2* (Figure 3a) was consistent with *in situ* hybridization, where it was shown that these genes are expressed from young buds through fruit development. RT-PCR expression of EeIND was not observed and *EeSHP2*, *EeFUL1*, and *EeFUL2* were only weakly expressed. *EeRPL* was expressed from young buds until fruit maturation. These RT-PCR data underscore the fact that the downstream DZ genes are expressed at low levels (Figure 3a; see Additional file 7: Figure S5d for lower cycle numbers) and, as a result, it is unsurprising that we were not able to observe expression of all these genes with *in situ* hybridization. No genes were expressed in leaves (Figure 3a).

### Valve margin pathway expression data in *Cakile lanceolata*

**Figure 4:**
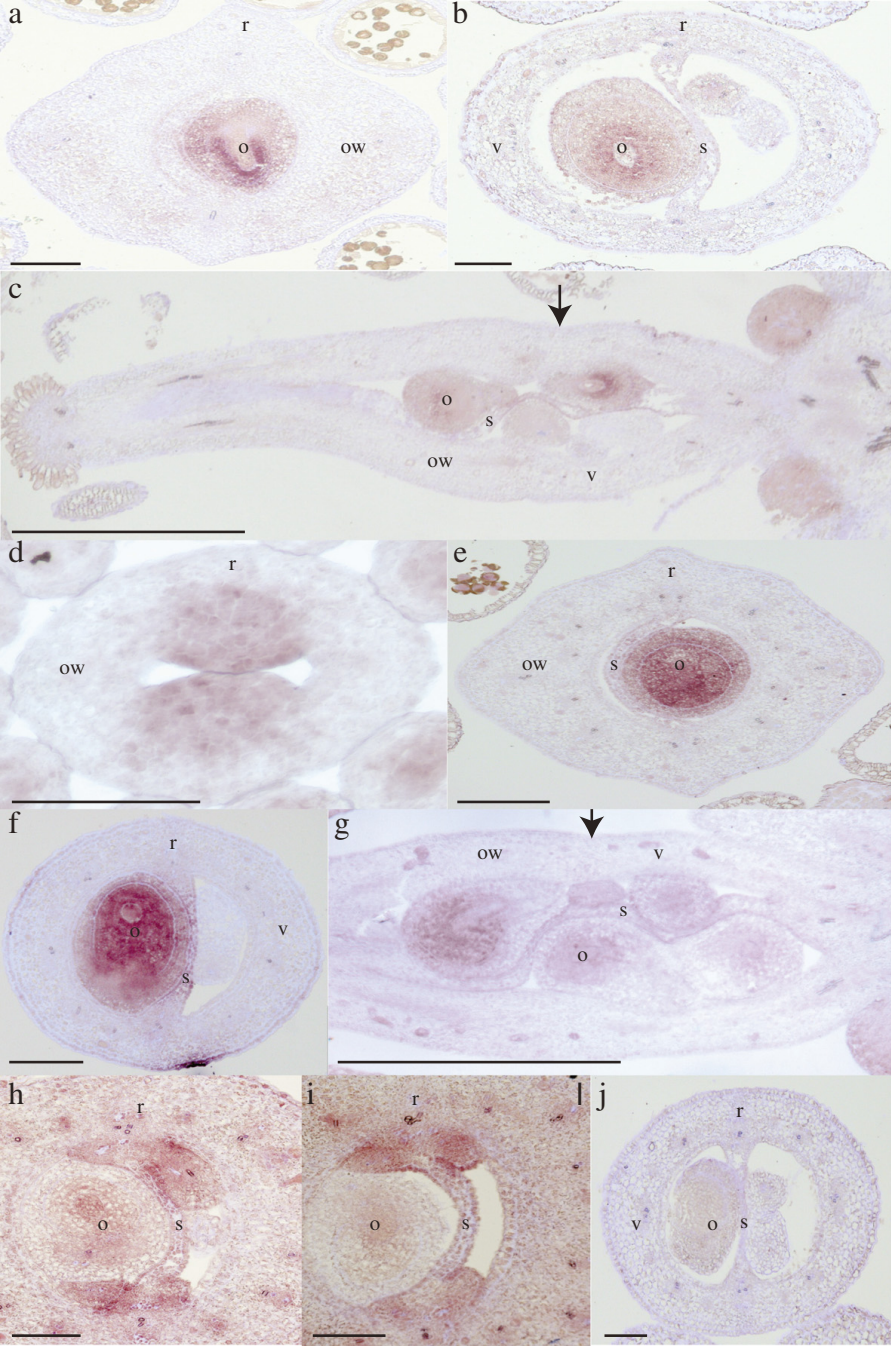
***In situ*hybridization expression of*ClSHP1*,*ClSHP2*, and*ClALC*during*Cakile lanceolata*carpel development. (a, b)** Transverse section through an old bud showed *ClSHP1* expression in the ovules of (**a**) distal and (**b**) proximal segments. (**c**) *ClSHP1* signal observed in the ovules, septum, and nectaries of an old bud, longitudinal section. (**d**) *ClSHP2* signal was observed in the developing placental tissue. (**e**-**g**) *ClSHP2* expression was observed in the ovules and septa of (**e**) distal and (**f**) proximal segments, transverse sections, and in longitudinal (**g**). (**h**, **i**) Transverse section through an older bud showed weak *ClALC* expression in septum, ovules, and funiculus in (**h**) proximal and (**i**) distal segments. (**j**) *ClALC* sense probe of an older bud. Arrows indicate the position of the joint. o, ovules; ow, ovary walls; r, replum; s, septum; v, valves. Scale bar: 100 μm (**a**, **b**, **d**, **e**, **f**, **h**, **i**, **j**), 500 μm (**c**, **g**).

We were able to characterize gene expression patterns of *ClSHP1*, *ClSHP2*, and *ClALC* with *in situ* hybridization, but no expression was detected for *ClFUL1*, *ClFUL2*, and *ClRPL* in these experiments. *ClSHP1* and *ClSHP2* had very similar expression domains. Later in development, expression of *ClSHP1* and *ClSHP2* was observed in the ovules with weaker expression in the septa (Figure 4a to c, e to g). Before carpel differentiation, *ClSHP2* was expressed in presumptive placental tissue (Figure 4d). Expression of *ClSHP1* was also detected in the nectaries (Figure 4c). No expression of *ClSHP1* or *ClSHP2* was observed in either the ovary wall or the joint. *ClALC* expression was detected after carpels had differentiated, where it was observed in the septum, ovules, and funiculus of both segments (Figure 4h to i). The signal was no longer observed once flowers reached anthesis (data not shown) and was never detected with sense probes (Figure 4j).

We also conducted RT-PCR on all identified loci to establish expression patterns that we were not able to observe with *in situ* hybridization (Figure 3b). Similar to results with *Erucaria*, we assessed expression based on 27 cycles (see Additional file 7: Figure S5d for lower cycle numbers). Consistent with *in situ* hybridization, *ClSHP1* and *ClSHP2* expression was observed in young buds through fruit maturation. *ClALC* expression was observed at earlier developmental stages (for example, young buds; Figure 3b) with RT-PCR than was observed with *in situ* hybridization. Contrary to what was observed with *in situ* hybridization, RT-PCR results indicated that *ClFUL1*, *ClFUL2*, and *ClRPL* were expressed across most developmental stages and tissue types with few exceptions. *ClRPL*, *ClSHP2*, and *ClALC* were not detected in leaves whereas *ClFUL1* was not detected in young buds.

## Discussion

**Figure 5:**
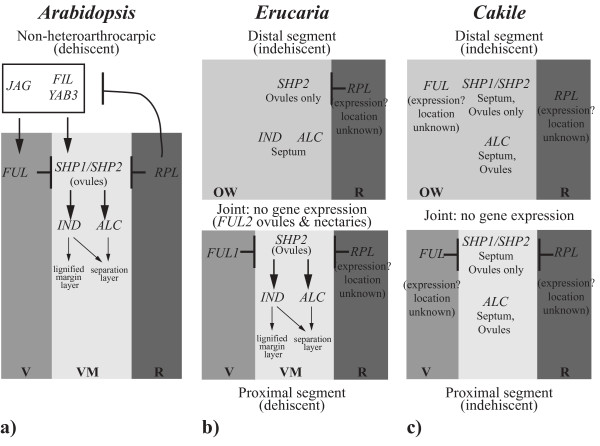
**Comparison of expression patterns of valve margin pathway genes between Arabidopsis,*Erucaria*, and*Cakile.*** (**a**) Valve margin pathway in Arabidopsis (modified from [11]). (**b**) Summary of valve margin pathway expression in distal (top) and proximal (bottom) of *Erucaria* based on *in situ* and RT-PCR data. (**c**) Summary of valve margin pathway expression in distal (top) and proximal (bottom) of *Cakile* based on *in situ* and RT-PCR data.

Although it has been hypothesized that indehiscence in some species of Brassicaceae may be due to relatively simple modifications in the valve margin genetic pathway [7,8], only four of the genes have been investigated in *Brassica* with emphasis placed on conservation of dehiscence rather than the evolution of indehiscence [23-25,45]. The current study represents the first time multiple members of this pathway have been investigated in species with marked indehiscence as well as novel segmentation. Our expression studies of 12 loci in *Erucaria* and *Cakile* have demonstrated both conservation and divergence in expression patterns of the fruit-patterning pathway (Figure 5). By examining the pathway in two species with different fruit morphologies, we provide insight into both the genetic basis of heteroarthrocarpy and the evolution of dispersal capabilities in Brassicaceae. 

### Dehiscence in *Erucaria*

In the critical valve dehiscence zone of the proximal segment of *Erucaria*, the expression domains of the *Erucaria SHP1/2*, *ALC*, and *IND* homologs are largely conserved relative to what has been observed in Arabidopsis and *Brassica*[14,17-19,46,47]. Outside the valve margin, some differences from Arabidopsis were found in expression of *EeIND* and *EeALC*[14], but the functional significance of these minor differences remains to be determined. Perhaps most intriguing is the expression of the *Erucaria FUL* homologs. In Arabidopsis, *FUL* is expressed throughout developing valve tissue and has a loss of function phenotype that exhibits defects in the differentiation and expansion of the valve, which is caused in part by an expansion of the *SHP1/2*, *IND*, and *ALC* expression domains [48]. The two copies of *FUL* from *Erucaria* have very different expression patterns from each other. *EeFUL1* is expressed in the mesocarp and end*b* layer of developing valve walls in the proximal segment (Figure 2l to n), implying possible conservation in the role of *EeFUL1* in limiting the expression of *EeSHP2*, *EeIND*, and Ee*ALC* to the valve margin. In contrast, *EeFUL2* is expressed in developing ovules and nectaries, but not in the ovary wall. Given that Arabidopsis *FUL* is also expressed in nectaries but not ovules [48], the novel *EeFUL2* expression pattern likely indicates a combination of neo- and subfunctionalization [49] or, alternatively, non-specific staining with our *EeFUL2* probes. Overall, however, the *EeFUL1* expression pattern is the most relevant in that it provides further evidence for the complex identity of the ovary wall in *Erucaria* and how heteroarthrocarpy relates to the typical silique of Arabidopsis. Hall *et al.* (2006) previously hypothesized that the valves of heteroarthrocarpic fruits are disassociated from the repla. This essentially means that although the replum and septum develop throughout the entire length of the fruit, only a basal region of the ovary wall differentiates as valve. If we treat *EeFUL1* expression as a kind of marker for valve identity, consistent with *FUL* function in Arabidopsis, these expression data would appear to confirm the hypothesis in showing that *FUL* homolog expression has contracted in *Erucaria* to encompass only the dehiscent portion of the ovary wall. This new domain seems to distinguish the functional valve region, which will dehisce and fall off, from the indehiscent portion of the ovary wall, which will form the persistent pericarp of the distal segment.

Unfortunately, no expression of *EeRPL* was observed in developing flowers and carpels with *in situ* hybridization, although RT-PCR indicates it is expressed in developing flowers and fruits (Figure 3a). This result was somewhat surprising as *RPL*, also known as *PENNYWISE* (*PNY*), *BELLRINGER* (*BLR*), and *VAAMANA* (*VAN*), is broadly expressed in developing Arabidopsis meristems due to an additional role in determining internode elongation [50,51]. More important to fruit development, in Arabidopsis, *RPL* negatively regulates *FIL*, *JAG*, and *YAB3*, three genes that redundantly function to activate *FUL* and *SHP*[11]. Because we have not been able to adequately capture *RPL* expression in this species with *in situ* hybridization, we cannot determine if there may be conservation between Arabidopsis and *Erucaria* on where the gene is expressed in developing carpels. Examination of *JAG*, *FIL*, and *YAB3* would also be informative. Fruits of *fil yab3* mutants lack a valve margin in the distal portion and have ectopic expression of valve margin in the proximal portion [11], which is somewhat reminiscent of heteroarthrocarpy.

### Indehiscence is correlated with loss of gene expression in the ovary wall

Indehiscence of the proximal segment of *Cakile* is associated with complete loss of gene expression of the valve margin pathway in the ovary wall and presumptive valve margin (Figure 5). Indehiscence of distal segments is also correlated with the absence of *SHP2*, *ALC*, and *FUL* expression in the ovary wall of *Erucaria* and the absence of *SHP1/SHP2* in *Cakile* (Figures 2, 4). This pattern is likely the result of this fruit segment having a completely novel developmental program when compared to Arabidopsis. In addition to redundant roles in promoting valve margin identity, *SHP1/SHP2* have partial redundancy in ovule development with *AGAMOUS* and *SEEDSTICK* in Arabidopsis [52]. As such, expression was maintained in the ovules of indehiscent segments of *Cakile* and *Erucaria* even when not observed in the carpel wall. A similar compelling pattern is observed with *EeALC*, *EeIND*, and *EeFUL1*. Expression of all three genes is observed in the presumptive valve margin or valve in dehiscent proximal segment, but not the indehiscent distal, thus highlighting the correlation of indehiscence with elimination of gene expression in the ovary wall. Our inability to visualize *ClFUL* and *ClRPL* expression in developing carpels, as suggested by RT-PCR data, means that we cannot assess their potential roles in the loss of *SHP* expression. Regardless, the current study suggests that indehiscence may be the result of the elimination of *SHP* expression in the developing fruit wall.

### Recruitment of valve margin pathway in joint abscission is unclear

No expression of any valve margin pathway gene was observed in the joint of either focal species, which is especially important when considering the abscising joint of *Cakile*. While it is possible that this pathway is simply not functioning in the joint, we must also consider the likelihood that difficulties in detecting expression of these loci have impaired our ability to assess their patterns. For example, it is possible that we have failed to capture homologs that may be expressed in the joint region. *ALC*, *IND*, and *RPL* have largely been characterized via reporter lines in Arabidopsis [14,18,20], suggesting that expression of these genes is challenging to determine even in model species. Thus we cannot eliminate with confidence the possibility that these genes are being expressed in the joint, which represents a modification in the distal portion of the valve margin [29]. Additionally, joint abscission occurs via the juxtaposition of non-lignified and lignified cells, which is an identical pattern to valve dehiscence [29]. This anatomy may reflect differential expression of an otherwise conserved valve margin pathway that could involve *ALC* and *IND* but not *SHP1/2* and *RPL*. The maintenance and expression of *ClFUL1* and *ClFUL2* in these indehiscent fruits is perplexing (Figure 3b), but suggest that these genes may be involved in joint abscission, even if they are not required for dehiscence. Alternatively, *FUL* may function to repress dehiscence zones in *Cakile*, similar to the role of *FUL* in Arabidopsis. However, we are currently unable to distinguish between these hypotheses due to technological issues with *in situ* hybridization.

## Conclusions

Heteroarthrocarpy and its variants have evolved multiple times across the tribe, implying lability in the underlying genetic pathway for this unusual fruit type [28]. Expression data from two variants of heteroarthrocarpy that represent a single evolutionary origin provide insight into how the valve margin pathway may have been modified. First, conservation in the expression patterns of genes promoting valve margin identity in the dehiscent segment of *Erucaria* was observed, suggesting these genes maintain the same functions as in Arabidopsis. In contrast, expression patterns of genes involved in positioning that pathway were different between *Erucaria* and Arabidopsis. These observations support anatomical data proposing that heteroarthrocarpy is the result of repositioning the valve margin [29], however they are not completely informative in precisely how the pathway has been repositioned. Additionally, indehiscence in all segments was characterized by the elimination of valve margin pathway and positioning genes. The absence of expression likely has a different developmental basis between distal and proximal segments as the ovary wall of distal segments does not differentiate into valves. Finally, the genetic basis of the joint remains elusive. Regardless, it is clear that modifications in expression patterns of the valve margin pathway are correlated with indehiscence and segmentation in *Cakile* and *Erucaria*.

## Abbreviations

*ALC*: *ALCATRAZ*; *BLR*: *BELLRINGER*; *Cl-*: *Cakile lanceolata-*; DZ: dehiscent zone; *Ee-*: *Erucaria erucarioides-*; end*b*: endocarp *b*; *FIL*: *FILAMENTOUS FLOWER*; *FUL*: *FRUITFULL*; *IND*: *INDEHISCENT*; *JAG*: *JAGGED*; *PNY*: *PENNYWISE*; *RPL*: *REPLUMLESS*; RT-PCR: reverse transcription-polymerase chain reaction; *SHP1*: *SHATTERPROOF1*; *SHP2*: *SHATTERPROOF2*; *VAN*: *VAAMANA*; *YAB3*: *YABBY3*.

## Competing interests

The authors declare that they have no competing interests.

## Authors’ contributions

JCH, EK, and KD conceived of a project, designed experiments, and developed drafts of manuscript. JCH and MA identified homologs and conducted *in situ* expression experiments. MA also carried out additional gene expression experiments, conducted phylogenetic analyses, generated figures, and developed manuscript. AJH conducted RT-PCR experiments and reviewed manuscript drafts. All authors read and approved the final manuscript.

## Supplementary Material

Additional file 1 **Table S1.** Primers used in the identification of homologs, *in situ*, and RT-PCR experiments. Click here for file

Additional file 2 **Table S2.** Taxa, loci, and accession numbers for sequences used in phylogenetic analyses (Additional file 3: Figures S1, Additional file 4: Figure S2, Additional file 5: Figure S3, and Additional file 6: Figure S4). Click here for file

Additional file 3 **Figure S1.** Neighbor joining tree of 104 genes from the AGAMOUS lineage, including SHATTERPROOF homologs identified from Cakile and Erucaria. Click here for file

Additional file 4 **Figure S2.** Neighbor joining tree of 37 genes from the APETALA1/FRUITFULL(FUL) lineage, including FUL homologs identified from Cakile and Erucaria. Click here for file

Additional file 5 **Figure S3.** Neighbor joining tree of 27 genes from the bHLH lineage, including ALCATRAZ and INDEHISCENT homologs identified from Cakile and Erucaria. Click here for file

Additional file 6 **Figure S4.** Neighbor joining tree of 26 genes from the BEL-like lineage, including REPLUMLESS homologs identified from Cakile and Erucaria. Click here for file

Additional file 7 **Figure S5.** Supplemental expression data of *in situ* hybridization of EeFUL1 and EeFUL2 and RT-PCR of all identified homologs at 20 and 22 cycles. Click here for file
