# Supplementary material for: Understanding the basis of a novel fruit type in Brassicaceae: conservation and deviation in expression patterns of six genes
Source: EvoDevo. 2012 Sep 3;3:20. doi: 10.1186/2041-9139-3-20 (PMC3503883; doi:10.1186/2041-9139-3-20)
Supplement: Additional file 1 — Table S1. Primers used in the identification of homologs, in situ, and RT-PCR experiments. [file 2041-9139-3-20-S1.pdf]

Table S1: Primer list.

Primers used to isolate homologs:

Locus	Name	Sequence 5'>3'
SHP1/2	For1: ATG3	ATGGSIMGIGGIAARATISARAT
	For2: AGf	ACIAAYMGICARGTIACITTYTG
	For3: SHP2fbp314	CAGAATCTGAACAGACACATTC
	Rev1: poly-T	CCGGATCCTCTAGAGCGGCCGC$(T)_{17}$
	Rev2: SHPrev	AAACAAGTTGIAGIGGIGGTTGGT
	Rev3: SHP1rev	CTCCGGATTCAATCTCGCACCTTGT
FUL	For1: AP1F	GTISARYTIAARMRRATIGARAAYAARAT
	For2: FULf	GAGCAYCARCTNGAYGCNGCNATHAARAG
	Rev1: poly-T	CCGGATCCTCTAGAGCGGCCGC$(T)_{17}$
	Rev2: FULr	GTNGGNCGYAACATCCANGCNGG
ALC	For1: ALCfor1	AGRAGRAGYAARATHAAYGARAARAT
	For2: ALCfor2	ATHCCNAAYTCNAAYAARACNGAYAA
	Rev: poly-T	CCGGATCCTCTAGAGCGGCCGC$(T)_{17}$
RPL	For1: RPLfor	GTNTAYAARAGRTAYARRCARTAYTA
	For2: RPLfor2	TAYTAYGARCARCTICARCGIGTIAT
	Rev1: poly-T	CCGGATCCTCTAGAGCGGCCGC$(T)_{17}$
	Rev2: RPLrev	TGDATYTCYTCNACCATNGGYTTCCA
IND	For1: INDfor1	CGNCGNGARAGRATHAGYCARAARAT
	For2: INDfor2	GTNCCNGGNGGNGCNAARATGGAYAC
	Rev: poly-T	CCGGATCCTCTAGAGCGGCCGC$(T)_{17}$

Primers used to generate in situ probes:

Locus	Forward primer 5'>3'	Reverse primer 5'>3'
ClSHP1	CAGAATTCAAATAGGCATATTG	CCGGATCCTCTAGAGCGGCCGC$(T)_{17}$
ClSHP2	CAGAATCTGAACAGACACATTC	TTAAACAAGTTGTAGAGGTG
EeSHP2	CAGAATCTGAACAGACACATTC	TTAAACAAGTTGTAGAGGTG
ClFUL1	GAGCAYCARCTNGAYGCNGCNATHAARAG	CCGGATCCTCTAGAGCGGCCGC$(T)_{17}$
ClFUL2	GAGCAYCARCTNGAYGCNGCNATHAARAG	CCGGATCCTCTAGAGCGGCCGC$(T)_{17}$
EeFUL1	GAGCAYCARCTNGAYGCNGCNATHAARAG	CCGGATCCTCTAGAGCGGCCGC$(T)_{17}$
EeFUL2	TCCAAAGCTTGGAGCAAGAGCATTAG	CCGGATCCTCTAGAGCGGCCGC$(T)_{17}$
ClALC	ATHCCNAAYTCNAAYAARACNGAYAA	CCGGATCCTCTAGAGCGGCCGC$(T)_{17}$
EeALC	ATHCCNAAYTCNAAYAARACNGAYAA	CCGGATCCTCTAGAGCGGCCGC$(T)_{17}$
ClRPL	GTGTATAAGAGGTATAAGCAGTATTA	GGAGCATGATGATCAGGGAATCC
EeRPL	GTGTATAAGAGGTATAAGCAGTATTA	GGAGCATGATGATCAGGGAATCC
EeIND	GTNCCNGGNGGNGCNAARATGGAYAC	CCGGATCCTCTAGAGCGGCCGC$(T)_{17}$

Primers used for RT-PCR:

Locus	Forward primer 5'>3'	Reverse primer 5'>3'
ClSHP1	CAGAATTCTAACAGGCATATAGTTGGGG	CTCCGGATTCAATCTCGCAACTTGT
ClSHP2	CTGAACAGACACATTCTTGGTGATTCTC	TTAAACAAGTTGTAGAGGTG
EeSHP2	CTGAACAGACACATTCTTGGTGATTCTC	TTAAACAAGTTGTAGAGGTG
ClFUL1	ATCAGCTCGCCGCTGCCATCAAGA	GAGGACGCACCGACGTTCTCT
ClFUL2	ATCAACTCCCTGCTGCTATCAAAA	GACGATGCATCTCCGTTCTCT
EeFUL1	ATCAGCTCGATGCTGCTATTAAGA	CGACGATGCAACGCCGTTCTCT
EeFUL2	TCCAAAGCTTGGAGCAAGAGCATTAG	CGATGCACCTCCGTCCCCAACT
ClALC	GACGGATAAAGCCTCAATGC	AGCGAAAATCTGACTTGGGA
EeALC	GACGGATAAAGCCTCAATGC	TGACATAGGATCATCTTCACCG
ClRPL	GTGTATAAGAGGTATAAGCAGTATTA	GGAGCATGATGATCAGGGAATCC
EeRPL	CAGCTGCAGGCGGTGATGGGCTCATTC	GCTTCCAAACCCTAACTCGTGCG
EeIND	CCCTTCTACCTGTGTCGGAGGTTACG	CCACCCATCGAGATGCAATATTA
ACTIN[1]	GATGGATCCTCCAATCCAGACACTGTA	GTATTGTGGACTCTGGTGATGGTGT

[1] Kramer EM, Holappa L, Gould B, Jaramillo MA, Setnikov D, Santiago PM: **Elaboration of B gene function to include the identity of novel floral organs in the lower eudicot *Aquilegia*.** *Plant Cell* 2007, **19:**750-766.
