# Supplementary material for: Understanding the basis of a novel fruit type in Brassicaceae: conservation and deviation in expression patterns of six genes
Source: EvoDevo. 2012 Sep 3;3:20. doi: 10.1186/2041-9139-3-20 (PMC3503883; doi:10.1186/2041-9139-3-20)
Supplement: Additional file 2 — Table S2. Taxa, loci, and accession numbers for sequences used in phylogenetic analyses (Additional file 3: Figures S1, Additional file 4: Figure S2, Additional file 5: Figure S3, and Additional file 6: Figure S4). [file 2041-9139-3-20-S2.pdf]

Table S2: Accession numbers for sequences used in phylogenetic analyses.

Taxon	Loci	GenBank Accession #
***SHATTERPROOF*-like genes accession number (see figure S1)**		
Agapanthus praecox Willd. subsp. *orientalis* (F.M. Leight.) F.M. Leight	*ApMADS2*	AB079260
Akebia quinata (Houtt.) Decne.	*AkqAG*	AY464107
Antirrhinum majus L.	*PLENA*	S53900
	FARINELLI	AJ239057
Aquilegia alpina L.	*AqaAG1*	AY464111
	AqaAG2	AY464110
Arabidopsis lyrata (L.) O'Kane & Al-Shehbaz subsp. *lyrata* (Fisher. & DC.) O'Kane & Al-Shehbaz	*ARALYDRAFT_486333*	XP_002876479
Arabidopsis lyrata (L.) O'Kane & Al-Shehbaz subsp. *petraea* (L.) O'Kane & Al-Shehbaz	*AlSHP1*	AAU82055
	AlSHP2	AAU82080
	ARALYDRAFT_321962	XP_002880005
Arabidopsis thaliana (L.) Heynh.	*AGAMOUS*	X53579
	SHP1/AGL1	M55550
	SHP2/AGL5	M55553
	STK/AGL11	U20182
Berberis gilgiana Fedde	*BgAG*	AY464106
Betula pendula Roth	*BpMADS6*	AJ252071
Brassica napus L.	*BnSHP1*	AY036062
	BnSHP2a	EU424342
	BnSHP2b	EU424343
Cakile lanceolata (Willdenow) O.E. Schulz	*ClSHP1*	XXXX
	ClSHP2	XXXX
Carica papaya L.	*Cp C-class*	ABR68012
Capsella bursa-pastoris (L.) Medik.	*CbSHP1b*	ACD76827
	CbSHP2b	ACD76825
	CbSTKb	ACD76823
Chrysanthemum x morifolium Ramat.	*CDM37*	AY173059
Chloranthus spicatus (Thunb.) Makino	*CsAG1*	AY464100
	CsAG2	AY464099
Clematis integrifolia L.	*CliAG1*	AY464113
	CliAG2	AY464112
Corylus avellana L.	*CaMADS1*	AF027376
Cucumis sativus L.	*CAG1*	AF022377
	CAG2	AF022378
	CAG3	AF022379
Cycas edentata de Laub.	*CyAG*	AF492455
Daucus carota L.	*DcMADS4*	AJ271150

Erucaria erucarioides Müll.Berol	*EeSHP2*	XXXX
Fragaria x ananassa hort.	*STAG1*	AF168468
Gerbera hybrida hort. cv. *'Terra Regina'*	*GAGA1*	AJ009722
	GAGA2	AJ009723
Ginkgo biloba L.	*GBM5*	AY114304
Gnetum gnemon L.	*GGM13*	AJ132219
Gossypium hirsutum L.	*GhMADS-2*	AF538966
	GhMADS-3	AY083173
Helianthus annuus L.	*HAM45*	AAO18228
Helleborus orientalis Lam.	*HoAG1a*	AY464109
	HoAG1b	AY464108
Hordeum vulgare L. subsp. *vulgare*	*HvAG1*	AF486648
	HvAG2	AF486649
Houttuynia cordata Thunb.	*HtcAG*	AY464096
Hyacinthus orientalis L.	*HAG1*	AF099937
Juglans regia L.	*JrAG*	AJ313088
Liquidambar styraciflua L.	*LAG*	AF103903
Lycopersicon esculentum L. Mill.	*TAG1*	L26295
	TAG11	AY098736
Magnolia praecocissima Koidz.	*MpMADS2*	AB050644
	MpMADS11	AB050653
Malus x domestica Borkh.	*MdMADS10*	AJ000762
	MdMADS14	AJ251117
	MdMADS15	AJ251118
Meliosma dilleniifolia (Wall. ex Wight & Arn.) Walp.	*MdAG1*	AY464098
	MdAG2	AY464105
Momordica charantia L.	*McAG*	AY178837
Nicotiana tabacum L.	*NAG1*	L23925
	NTPLE36	U63163
Nymphaea sp. EMK-2003	*NymAG1*	AY464104
	NymAG2	AY464103
	NymAG3	AY464102
Oryza sativa L.	*OsMADS3*	L37528
	OsMADS13	AF151693
Panax ginseng C.A. Mey.	*GAG2*	Z46612
Petunia x hybrida hort. ex Vilm.	*FBP6*	X68675
	FBP7	X81651
	FBP11	X81852
	pMADS3	X72912
Picea abies (L.) H. Karst	*dal2*	X79280
Picea mariana (Mill.) Britton, Sterns & Poggenb.	*SAG1a*	U69482
Pinus radiata D. Don	*AF023615*	AF234617
Phalaenopsis equestris (Schauer) Reichb. f.	*PeMADS1*	AF023615
Phytolacca americana L.	*PhaAG1*	AY464093

	PhaAG2	AY464118
Populus balsamifera L. subsp. trichocarpa (Torr. & A. Gray) Brayshaw	*PTAG1*	AF052570
	PTAG2	AF052571
Ranunculus ficaria L.	*RfAG1*	AY464114
	RfAG2	AY464115
Rosa rugosa Thunb.	*MASAKOC1*	AB025644
	MASAKOD1	AB025643
Rumex acetosa L.	*RaP1*	X89107
Sanguinaria canadensis L.	*ScAG*	AY464097
Saruma henryi Oliv.	*SrhAG*	AY464101
Saxifraga careyana A. Gray	*SxcAG1*	AY464117
	SxcAG2	AY464116
Silene latifolia Poir. subsp. *alba* (Mill.) Greuter & Burdet	*SLM1*	X80488
Thalictrum dioicum L.	*ThdAG1*	AY464095
	ThdAG2	AY464094
Triticum aestivum L.	*WAG*	AB084577
Vitis vinifera L.	*VvMADS1*	AF265562
	VvMADS5	AF373604
Zea mays L.	*ZAG1*	L18924
	ZAG2	L18925
	ZMM1	X81199
	ZMM2	X81200
	Zmm23	AJ430637
	Zmm25	AJ430639
***FRUITFULL*-like genes accession number (see Fig. S2)**		
Aquilegia sp.	*AqaAP1*	?
Antirrhinum majus	*euFUL*	AAP83363
	SQUA	X63701
Arabidopsis thaliana	*FUL/AGL8*	AAA97403
	AP1	Z16421
	CAL	L36925
Betula pendula	*BpMADS3*	X99653
	BpMADS5	X99655
Brassica oleracea L.	*BoCAL*	L36926
Brassica oleracea L. var. *botrytis* L.	*BOAP1*	Z37968
	FUL-a	CAD47849
	FUL-b	CAD47850
	FUL-c	CAD47851
	FUL-d	CAD47852
Brassica oleracea L. var. *italica* Plenck	*boiAP1*	AAB08875
	boiCAL	AAB08878
Brassica rapa L. subsp. *pekinensis* (Lour.) Hanelt	*pCAL*	AJ251300

Cakile lanceolata	*ClFUL1*	XXXX
	ClFUL2	XXXX
Corylopsis sinensis Hemsl.	*CsFUL*	AY306147
Erucaria erucarioides	*EeFUL1*	XXXX
	EeFUL2	XXXX
Gerbera hybrida cv. *'Terra Regina'*	*GSQUA1*	AJ009727
Lolium perenne L.	*LpMADS1*	AY198326
	LpMADS2	AY198327
Lycopersicon esculentum	*TM4*	Q40170
Malus domestica	*MdMADS2*	U78948
	MdMADS5	AJ000759
Phytolacca americana	*PaFUL*	AY306163
Silene latifolia subsp. *alba*	*SLM4*	X80491
	SLM5	X80492
Sinapis alba L.	*SaMADSB*	U25695
	SaMADSC-2	AF109403
Solanum commersonii Dunal	*SCM1*	AF002666
Sorghum bicolor (L.) Moench	*SbMADS2*	U32110
Triticum aestivum	*TaMADS#11*	AB007504
Zea mays	*ZAP1*	L46400
**BHLH-like genes accession number (see Fig. S3)**		
Arabidopsis lyrata subsp. *lyrata*	*ARALYDRAFT_496889*	XP_002865027
	EDA33	XP_002872969
Arabidopsis thaliana	*ALC*	Q9FHA2
	HEC3	Q9LXD8
	IND	NP_191923
	PIF3	O80536
	PIF4	BAH20328
	SPT	NP_568010
Brassica oleracea	*BolC.IND.a*	ADC68234
Brassica rapa subsp. *pekinensis*	*BraA.IND.a*	ADC68235
Cakile lanceolata	*ClALC*	XXXX
Catharanthus roseus (L.) G. Don	*MYC5*	ACM41588
Erucaria erucarioides	*EeALC*	XXXX
	EeIND	XXXX
Fragaria x ananassa	*FabHLH*	AAV33474
Lepidium campestre (L.) W.T. Aiton	*LpIND*	ACR15952
Lotus japonicus (Regel) K. Larse	*LjBHLH9*	ACN21633
	LjBHLH12	ACN21636
	LjBHLH13	ACN21637
Oryza sativa Japonica Group	*OsSPT*	BAD67851
Prunus persica (L.) Batsch	*PpALC/SPT*	ADG56590
Ricinus communis L.	*RCOM_1461320*	XP_002517254
	RCOM_1409990	XP_002523613

	VvHEC3	NP_001131794
Vitis vinifera	*LOC100245665*	XM_002277930
Zea mays	*LOC100193167*	NM_001138322
	ZM_BFc0135I21	BT065018
***REPLUMLESS*-like genes accession number (see Fig. S4)**		
Arabidopsis lyrata subsp. *lyrata*	*ARALYDRAFT_486980*	XP_002870947
Arabidopsis thaliana	*BEL1*	NM_123506
	BEL10	NP_173400
	BLH4/SAW2	NP_179956
	BLH6	NP_195187
	BLH7	NP_179233
	BLH8/PNF	NP_180366
	BLH2_ARATH	Q9SW80
	BLH3_ARATH	Q8S897
	BLH5_ARATH	Q9FWS9
	RPL/BLH9_ARATH	Q9LZM8
Cakile lanceolata	*ClRPL*	XXXX
Erucaria erucarioides	*EeRPL*	XXXX
Hordeum vulgare subsp. *vulgare*	*JUBEL1*	AAK38645
	JUBEL2	AAK38646
Malus x domestica	*Mdh1*	AAF43095
Oryza sativa Indica Group	*qSH-1*	BAB85942
Ricinus communis	*RCOM_1156630*	XP_002529855
Sorghum bicolor	*SORBIDRAFT_09g022270*	XP_002439906
Solanum etuberosum Lindl.	*BEL5*	ACD39468
	BEL30	ACD39463
Solanum tuberosum L.	*StBel13*	AAN03623
	StBel22	AAN03625
Triticum aestivum	*WBLH3*	BAJ04689
Vitis vinifera	*VITISV_024375*	CAN73410
Zea mays	*ZmBHL30*	NP_001147963
