# Supplementary material for: Understanding the basis of a novel fruit type in Brassicaceae: conservation and deviation in expression patterns of six genes
Source: EvoDevo. 2012 Sep 3;3:20. doi: 10.1186/2041-9139-3-20 (PMC3503883; doi:10.1186/2041-9139-3-20)
Supplement: Additional file 3 — Figure S1. Neighbor joining tree of 104 genes from the AGAMOUS lineage, including SHATTERPROOF homologs identified from Cakile and Erucaria. [file 2041-9139-3-20-S3.pdf]

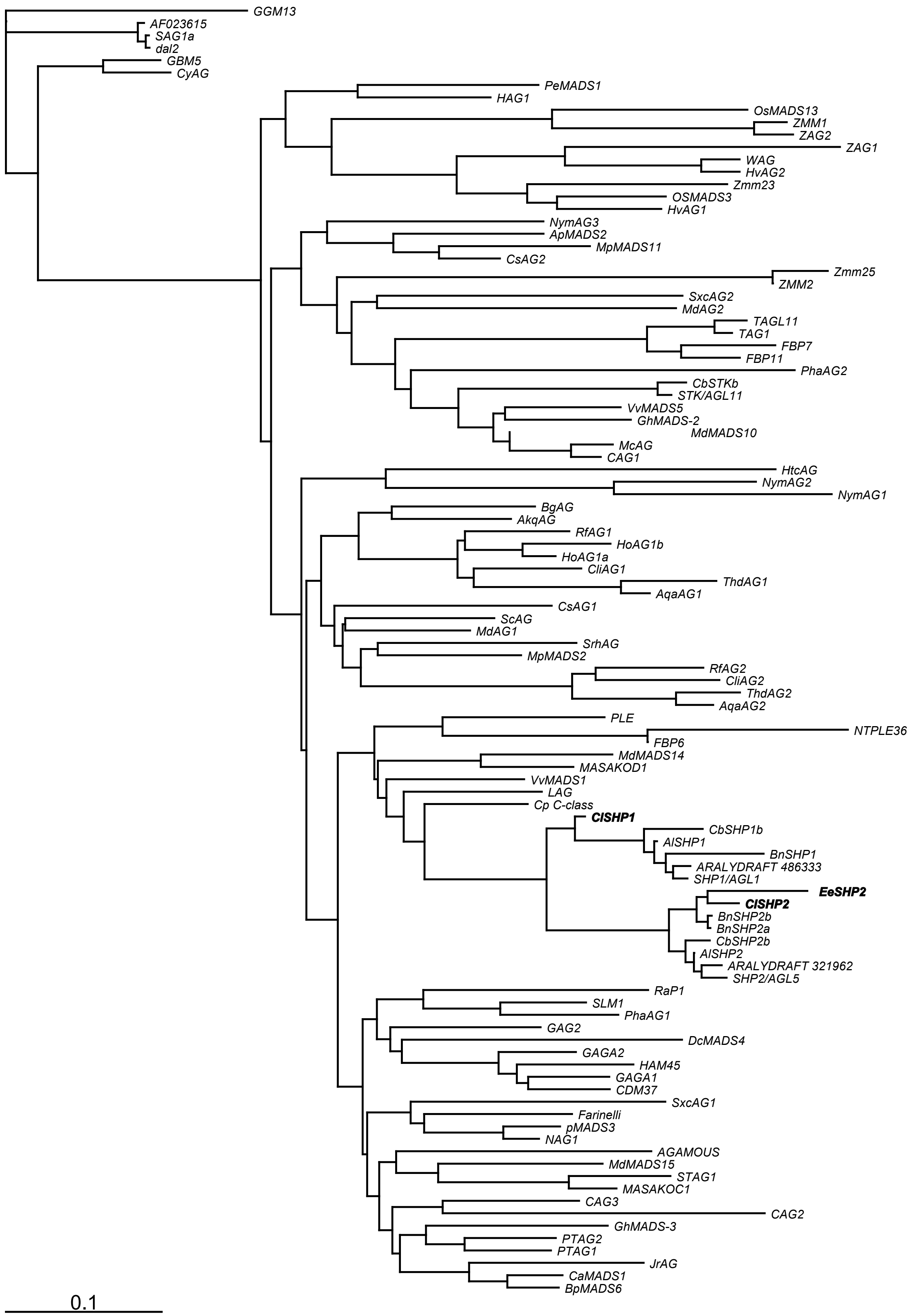


**Figure S1**. Neighbor joining tree of 105 *AGAMOUS*-like genes, including *SHATTERPROOF* homologs identified from *Cakile* (*ClSHP1* and *ClSHP2*, in bold) and *Erucaria (EeSHP2*, in bold). Taxa names and GenBank accession numbers are provided in Table S2.
