# Supplementary material for: Understanding the basis of a novel fruit type in Brassicaceae: conservation and deviation in expression patterns of six genes
Source: EvoDevo. 2012 Sep 3;3:20. doi: 10.1186/2041-9139-3-20 (PMC3503883; doi:10.1186/2041-9139-3-20)
Supplement: Additional file 4 — Figure S2. Neighbor joining tree of 37 genes from the APETALA1/FRUITFULL(FUL) lineage, including FUL homologs identified from Cakile and Erucaria. [file 2041-9139-3-20-S4.pdf]

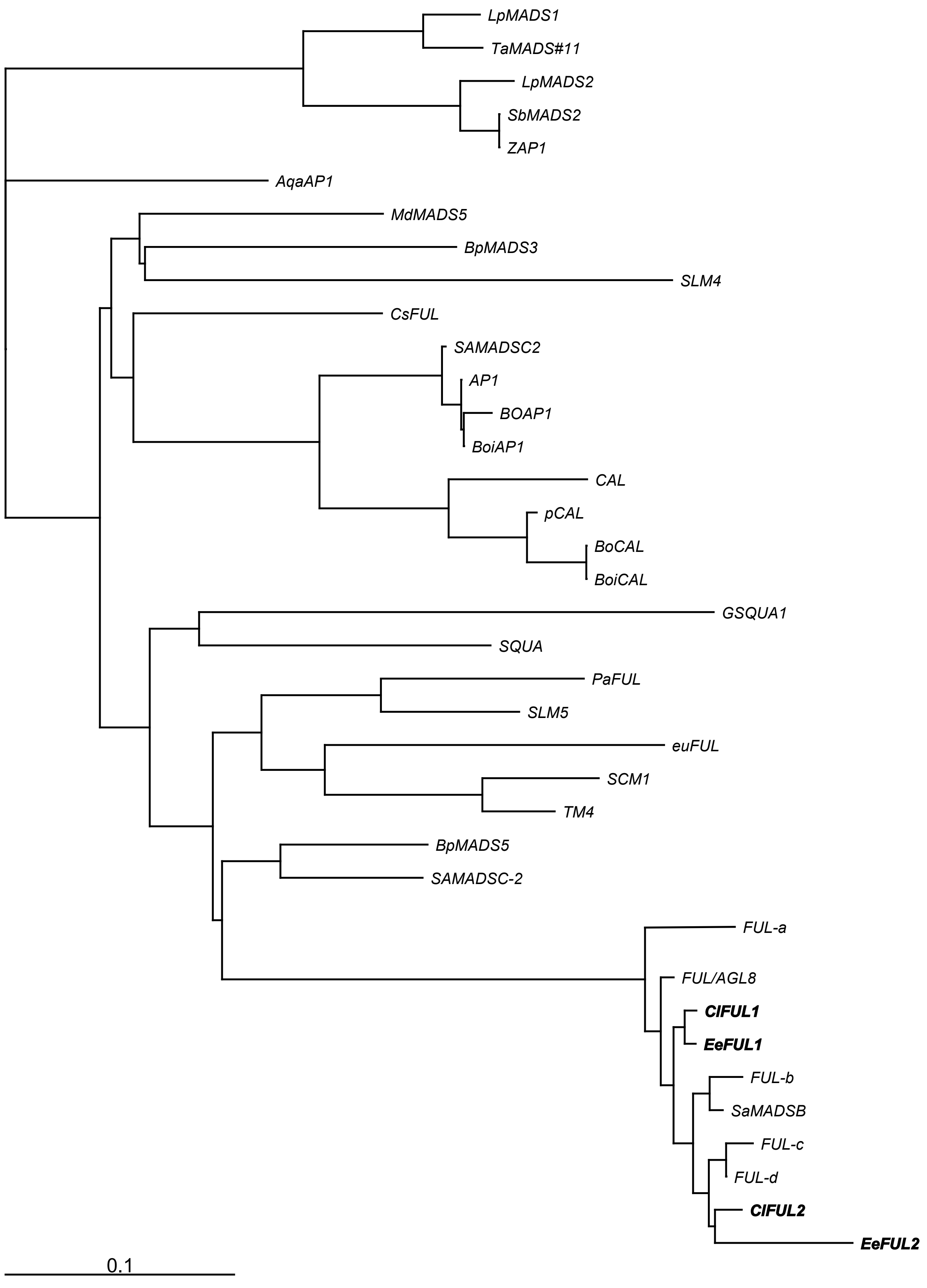

Figure S2. Neighbor joining tree of 37 *APETALA1/FRUITFULL*(*FUL*)-like genes, including *FUL* homologs identified from *Cakile* and *Erucaria* (*ClFUL1*, *ClFUL2*, *EeFUL1*, and *EeFUL2* in bold). Taxa names and GenBank accession numbers are provided in Table S2.
