# Supplementary material for: Understanding the basis of a novel fruit type in Brassicaceae: conservation and deviation in expression patterns of six genes
Source: EvoDevo. 2012 Sep 3;3:20. doi: 10.1186/2041-9139-3-20 (PMC3503883; doi:10.1186/2041-9139-3-20)
Supplement: Additional file 5 — Figure S3. Neighbor joining tree of 27 genes from the bHLH lineage, including ALCATRAZ and INDEHISCENT homologs identified from Cakile and Erucaria. [file 2041-9139-3-20-S5.pdf]

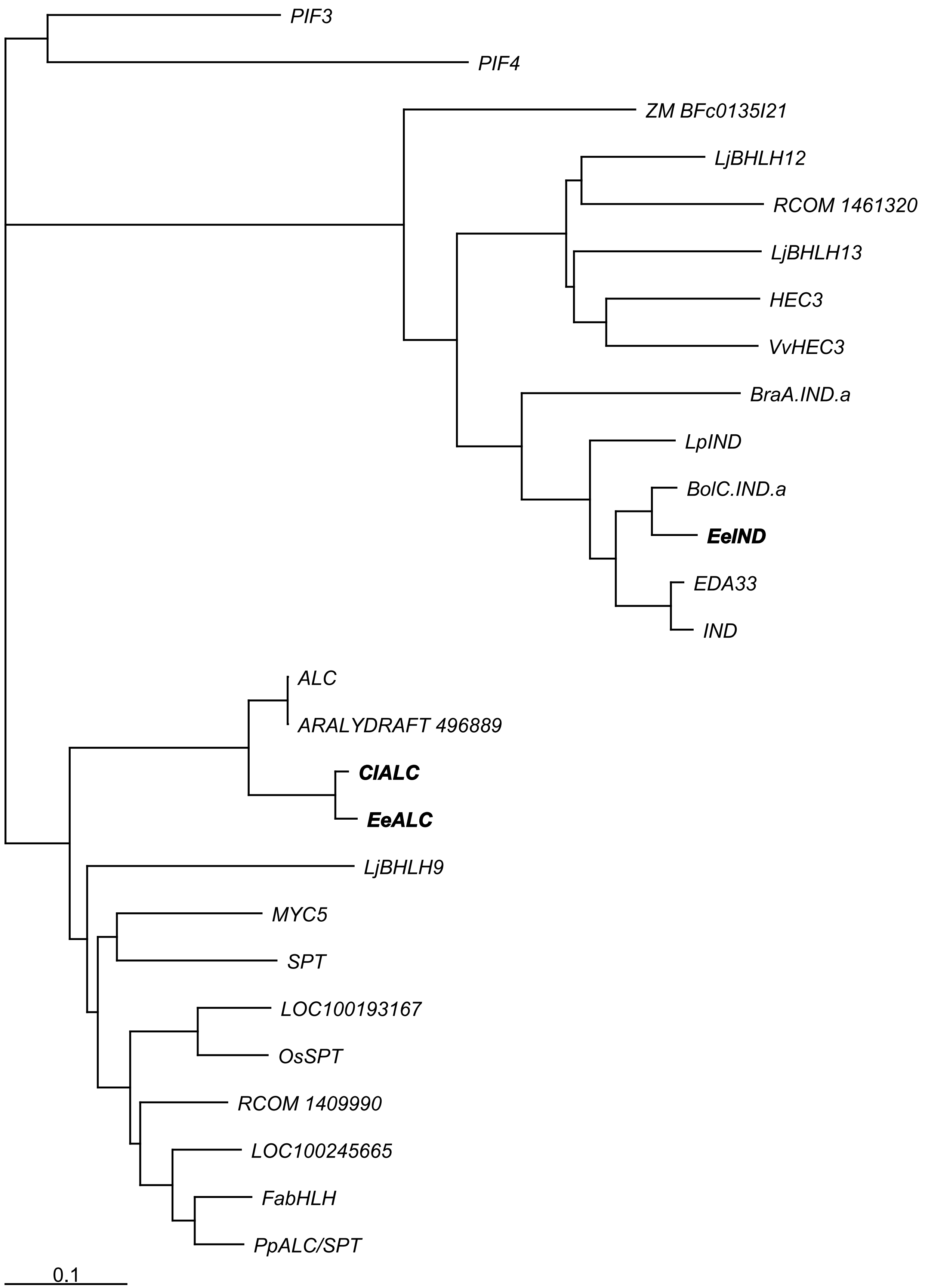

Figure S3. Neighbor joining tree of 27 bHLH-like genes (*ALC*, *HEC3*, *IND*, and *SPT*-like genes), including homologs identified from *Cakile* and *Erucaria* (*ClALC*, *EeALC*, and *ClIND,* in bold). Taxa names and GenBank accession numbers are provided in Table S2.
