# Supplementary material for: Understanding the basis of a novel fruit type in Brassicaceae: conservation and deviation in expression patterns of six genes
Source: EvoDevo. 2012 Sep 3;3:20. doi: 10.1186/2041-9139-3-20 (PMC3503883; doi:10.1186/2041-9139-3-20)
Supplement: Additional file 6 — Figure S4. Neighbor joining tree of 26 genes from the BEL-like lineage, including REPLUMLESS homologs identified from Cakile and Erucaria. [file 2041-9139-3-20-S6.pdf]

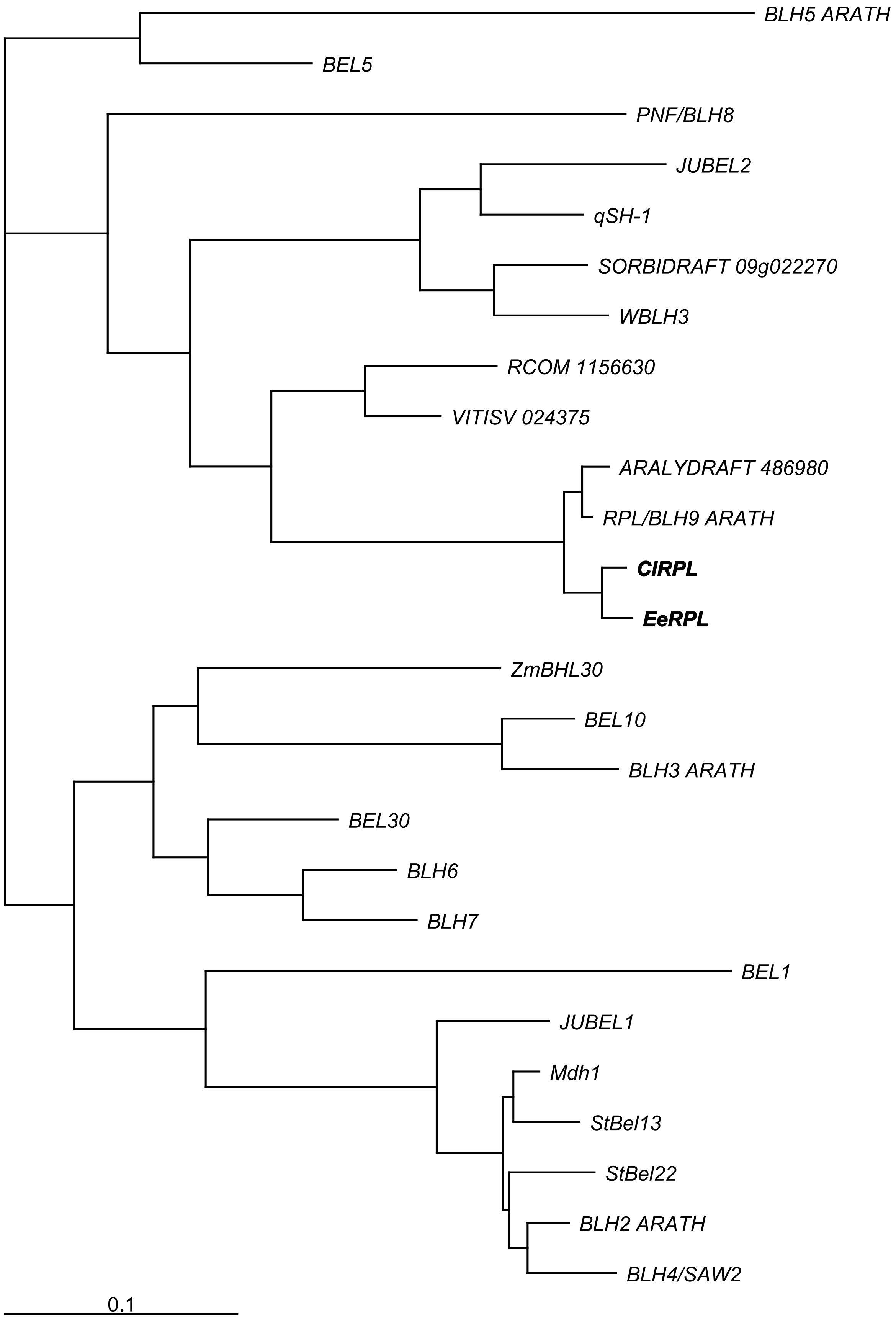

Figure S4: Neighbor joining tree of 26 BEL-like genes, including homologs identified from *Cakile* and *Erucaria* (*ClRPL* and *EeRPL* in bold). Taxa names and GenBank accession numbers are provided in Table S2.
