# Supplementary material for: Understanding the basis of a novel fruit type in Brassicaceae: conservation and deviation in expression patterns of six genes
Source: EvoDevo. 2012 Sep 3;3:20. doi: 10.1186/2041-9139-3-20 (PMC3503883; doi:10.1186/2041-9139-3-20)
Supplement: Additional file 7 — Figure S5. Supplemental expression data of in situ hybridization of EeFUL1 and EeFUL2 and RT-PCR of all identified homologs at 20 and 22 cycles. [file 2041-9139-3-20-S7.pdf]

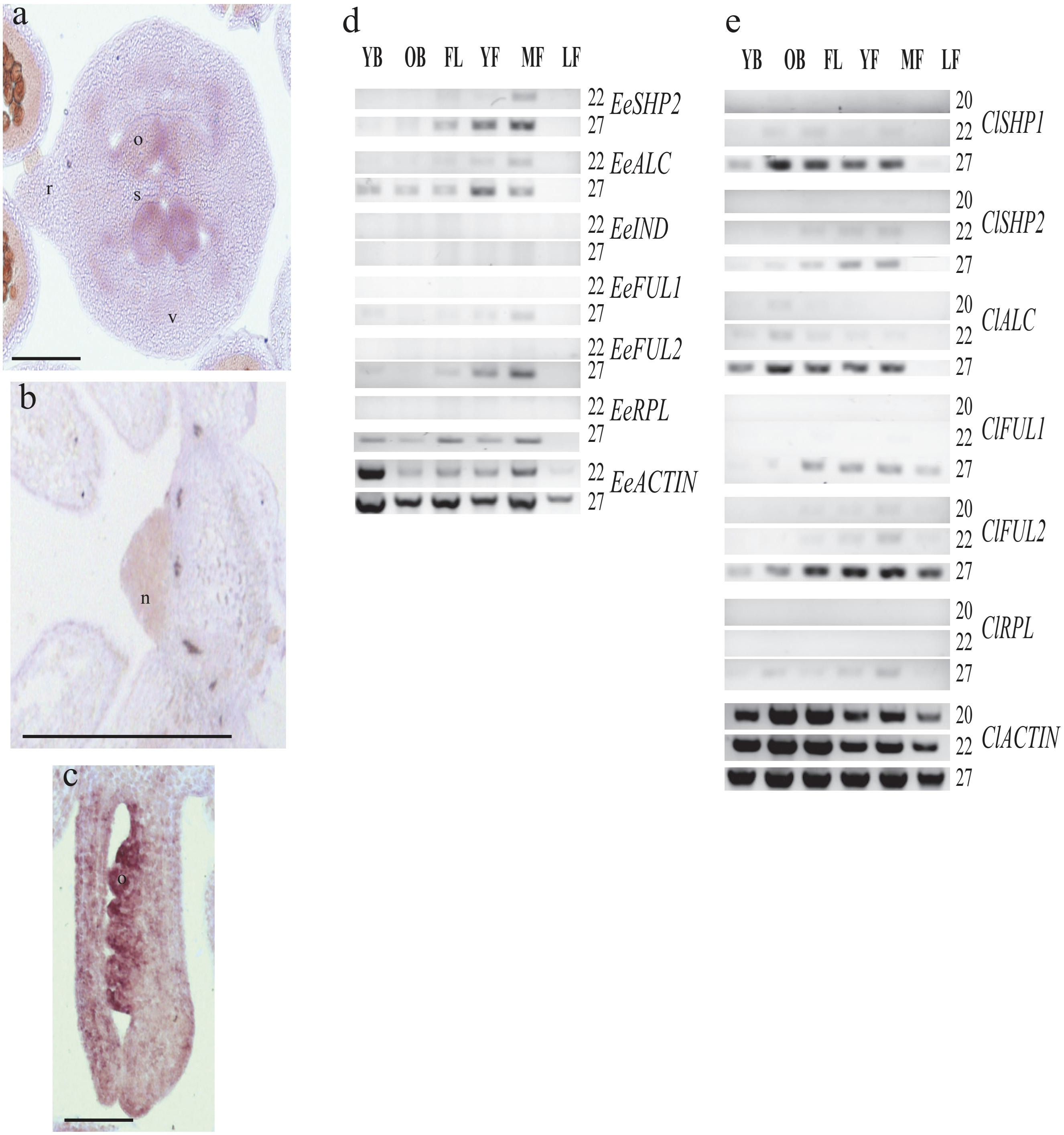

Figure S5.Additional expression data. (a-c) In situ hybridization expression of *EeFUL1* and *EeFUL2* during *Erucaria erucarioides* carpel development.(a) Transverse section through proximal segment of an old bud showed *EeFUL1* expression in inner valves and ovules. (b) *EeFUL1* was later expressed in nectaries, longitudinal section. (c) Longitudinal section through a young bud showed *EeFUL2* signal in ovule primordia. (d-e) Different cycle durations of RT-PCR on (d) *Erucaria erucarioides* and (e) *Cakile lanceolata.* n:nectary, o: ovules, ow: ovary walls, r: replum, s: septum, v: valves. Scale bar: 100 µm (a, c), 500 µm (b).
